# Auto-Weighted Multi-View Discriminative Metric Learning Method With Fisher Discriminative and Global Structure Constraints for Epilepsy EEG Signal Classification

**DOI:** 10.3389/fnins.2020.586149

**Published:** 2020-09-29

**Authors:** Jing Xue, Xiaoqing Gu, Tongguang Ni

**Affiliations:** ^1^Department of Nephrology, The Affiliated Wuxi People's Hospital of Nanjing Medical University, Wuxi, China; ^2^School of Computer Science and Artificial Intelligence, Changzhou University, Changzhou, China

**Keywords:** metric learning, multi-view learning, auto-weight, EEG signal classification, epilepsy

## Abstract

Metric learning is a class of efficient algorithms for EEG signal classification problem. Usually, metric learning method deals with EEG signals in the single view space. To exploit the diversity and complementariness of different feature representations, a new *a*uto-weighted *m*ulti-view *d*iscriminative *m*etric *l*earning method with Fisher discriminative and global structure constraints for epilepsy EEG signal classification called AMDML is proposed to promote the performance of EEG signal classification. On the one hand, AMDML exploits the multiple features of different views in the scheme of the multi-view feature representation. On the other hand, considering both the Fisher discriminative constraint and global structure constraint, AMDML learns the discriminative metric space, in which the intraclass EEG signals are compact and the interclass EEG signals are separable as much as possible. For better adjusting the weights of constraints and views, instead of manually adjusting, a closed form solution is proposed, which obtain the best values when achieving the optimal model. Experimental results on Bonn EEG dataset show AMDML achieves the satisfactory results.

## Introduction

Epilepsy is characterized by an unexpected seizure periodicity, where brain temporary dysfunction is caused by abnormal discharge of neurons (Kabir and Zhang, 2016; Gummadavelli et al., 2018; Li et al., 2019). During the seizure, motor dysfunction, intestinal and bladder dysfunction, loss of consciousness, and other cognitive dysfunction often occur. Since the occurrence of epilepsy is often accompanied by changes in spatial organization and temporal dynamics of brain neural neurons, many brain imaging methods are used to reveal abnormal changes in brain neural neurons caused by epilepsy. EEG signal is an important signal to record the activity of neurons in the brain. It uses electrophysiological indicators to record the changes in the electrical wave of the cerebral cortex generated during brain activity. It is the overall reflection of the activity of brain neurons in the cerebral cortex. Many clinical studies have shown that due to abnormal discharge of brain neurons, epilepsy-specific waveforms, such as spikes and sharp waves, appear during or shortly before the onset of seizures, so identifying EEG signals is an effective detection of epilepsy method. Clinically, the detection of seizures based on EEG signals mainly relies on the personal experience of doctors. However, modern EEG recorders can generate up to 1,000 data points per second, and the standard recording process can last for several days. This procedure will make manual screening require a lot of physical and mental exhaustion, and after a long period of observation, the doctor's judgment is easily affected by fatigue.

With the gradual development of smart healthcare, more and more machine learning algorithms are applied to the detection of epilepsy of EEG signals (Jiang et al., 2017a; Juan et al., 2017; Usman and Fong, 2017; Richhariya and Tanveer, 2018; Cury et al., 2019). In the view of machine learning, the EEG signal recognition contains two stages: feature extraction and classification method. The commonly used feature extraction methods for EEG signals are time-domain feature extractions and frequency-domain feature extractions (Srinivasan et al., 2005; Tzallas et al., 2009; Iscan et al., 2011). Since the original EEG signal is the time series signal, the time-domain feature extractions are generally based on the original EEG signal; then, the relevant statistics of the time series are calculated, and the epilepsy EEG features are extracted, using the kernel principal component analysis (KPCA) (Smola, 1997). The frequency-domain features are to transform the original EEG signal in the time domain to the frequency domain and then extract the relevant frequency-domain features as EEG features (Griffin and Lim, 1984). Although these feature extraction methods provide good performance in some practical applications, there is no feature extraction method that can be applied to all application scenarios. EEG signals are generated by numerous brain neuron activities. Due to the non-linear and non-static nature of EEG signals, how to extract effective features is still an important challenge. For those reasons, the multiple feature based multi-view learning concept has become a hot topic in EEG signal classification (Yuan et al., 2019; Wen et al., 2020). Different from using the single feature type, the multi-view learning method can comprehensively use a set of data features obtained from multiple ways or multiple levels. Each view of the data features may contain specific information not available in other views. Specifically, these independent and diverse features can be extracted from time-domain, frequency-domain, and multilevel features of signals. Appropriately designed multi-view learning can significantly promote the performance of EEG signal classification. For example, Spyrou et al. (2018) proposed a multiple features-based classifier to use spatial, temporal, or frequency EEG data. This classifier performs dimensionality reduction and rejects components through evaluating the classification performance. Two multi-view Takagi–Sugeno–Kang fuzzy systems for epileptic EEG signals classification are proposed in Zhou et al. (2019) and Jiang et al. (2017b), respectively. The former fuzzy system is developed in a deep view-reduction framework, and the latter fuzzy system is developed in a multi-view collaborative learning mechanism.

Besides the multi-view learning, classification algorithm is very important for EEG signal classification. One of the recent trends is the metric learning method. Metric learning method learns a more suitable distance measurement criterion in the feature space from the training data. Metric learning can be used for specific tasks, such as classification and clustering, so as to more accurately represent the similarity between samples. Different from traditional Euclidean distance, such as nearest-neighbors classifiers and K-means, metric learning aims to find the appropriate similarity measures between data pairs to maintain the required distance structure (Cai et al., 2015; Wang et al., 2015; Lu et al., 2016). The appropriately distance metrics can provide a good measure of the similarity and dissimilarity between different samples. For example, Liu et al. (2014) developed a similarity metric-learning in the process of EEG P300 wave recognition. Compared with traditional Euclidean metric, the proposed global Mahalanobis distance metric shows the better discriminative representation. Phan et al. (2013) proposed a metric learning method using the global distance metric from labeled examples. This method successfully applied on single-channel EEG data for sleep staging and does not need artifact removal or boostrapping preprocessing steps. Alwasiti et al. (2020) proposed a deep metric learning model and tested it for motor imagery EEG signals classification. The experimental results show that the proposed deep metric learning model can converge with very small number of training EEG signals.

Inspired by the distance metric and multi-view learning, we present a new auto-weighted multi-view discriminative metric learning method with Fisher discriminative and global structure constraints for EEG signal classification called AMDML. To better exploit the correlation and complementary data features of multiple views, both the Fisher discriminative constraint and global structure constraint are adopted in the construction process of the distance metric matrix. In such common metric space, the intraclass EEG signals are compact, and interclass EEG signals are separable as much as possible. Simultaneously, an auto-weighted learning strategy is developed to automatically adjust constraint and view weights during the model learning process. The contributions of our work are as follows: (1) Both Fisher discriminative and global structure information of multiple view data features are considered in the multi-view metric learning model with the high discriminative performance; (2) in the optimization process, the constraint and view weights can be adjusted auto-weighted by the closed form solution, instead of adjusted manually. Thus, the constraints balance and multiple view collaboration can be optimized; and (3) the experimental results on Bonn EEG dataset justify the applicability of AMDML for EEG signal classification.

## Related Work

### Metric Learning

Here, we introduce the baseline method in the study. Xing et al. (2003) proposed a distance metric considering side-information (DMSI) method. Using the given similar and dissimilar pairs of samples, DMSI learns a good distance metric to identify the “similar” relationship between all pairs of samples so that similar pairs are close and dissimilar pairs are separated. Let *S* and *D* be two sets of pair as

(1) $$\begin{array}{l} S=\left\{\left(x_{i},x_{j}\right)|x_{i}\;and\;x_{j}\;are\;similar\right\}, \end{array}$$

(2) $$\begin{array}{l} D=\left\{\left(x_{i},x_{j}\right)|x_{i}\;and\;x_{j}\;are\;dissimilar\right\}. \end{array}$$

The optimization problem of DMSI is represented as

(3) $$\begin{array}{l} \begin{array}{l} \underset{M}{\min}\overset{\;}{\underset{\left(x_{i},x_{j}\right)\in S}{\sum }}\|x_{i}-x_{j}{\|}_{M}^{2}\\ s.t.\;\overset{\;}{\underset{\left(x_{i},x_{j}\right)\in D}{\sum }}\|x_{i}-x_{j}{\|}_{M}^{\;}\geq 1,\\ \;M\geq 0. \end{array} \end{array}$$

A key point in DMSI is that all samples that are not clearly identified as similar are dissimilar. In addition, metric learning tries to find an appropriate measurement to preserve the distance structure. The distance metric considers a positive semidefinite matrix ***M***, and $\|x_{i}-x_{j}{\|}_{M}^{\;}$ is induced as a Mahalanobis distance

(4) $$\begin{array}{l} \|x_{i}-x_{j}{\|}_{M}^{\;}=\sqrt{{\left(x_{i}-x_{j}\right)}^{T}M\left(x_{i}-x_{j}\right)} \end{array}$$

When the learned ***M*** is a diagonal matrix, **Equation (3)** can be solved by the Newton–Raphson method; when ***M*** is a full matrix, **Equation (3)** can be solved by an iterative optimization algorithm with gradient ascent and iterative projection strategies.

### Bonn EEG Dataset

**Figure 1:**
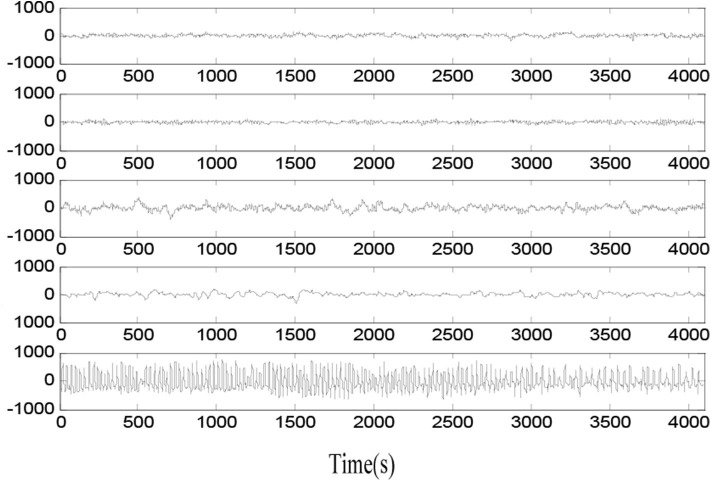
Original epileptic EEG signals in five groups.

The EEG signal data in the experiment is from the website of the Bonn University, Germany (Tzallas et al., 2009). The Bonn EEG dataset contains five groups of EEG signal sets called as groups A–E. The example samples in groups A–E are shown in Figure 1. Each EEG data group consists of 100 single-channel EEG signal segments of 23.6 s and 173.6 Hz rate. The basic information of five groups is listed in Table 1. EEG signal data in groups A and B is sampled from five healthy volunteers, and EEG signal data in groups C–E is sampled from five patients at different states of epileptic seizure.

**TABLE 1:** The basic information of EEG data groups of A-E.

**Group**	**Basic information**
A	EEG signals of healthy volunteers in awaken state with eyes open
B	EEG signals of healthy volunteers in awaken state with eyes closed
C	EEG signals of patients in hippocampal formation of the opposite hemisphere of brain
D	EEG signals of patients in epileptogenic zone during periodic lulls
E	EEG signals of patients during seizure activity

## Proposed Method

### Objective Function

After collecting a set of EEG signals, we obtain *N* samples presented as ${\left\{x_{i}^{\;},l_{i}\right\}}_{i=1}^{N}$, where *l*_*i*_ is the class label of sample $x_{i}^{\;}$. According to the label information, we construct two sets of sample pairs such that $X_{s}=\left\{\left(x_{i},x_{j}\right)|l_{i}^{\;}\;=l_{j}^{\;}\right\}$ is the intraclass sample set and $X_{d}=\left\{\left(x_{i},x_{j}\right)|l_{i}^{\;}\neq \;l_{j}^{\;}\right\}$ is the interclass sample set. Then, generating multiple view of data samples, we obtain ${\left\{x_{i}^{m}\right\}}_{m=1}^{M}$ from *M* different view features of each sample, where $x_{i}^{m}$ is the *m*th view of sample $x_{i}^{\;}$. For discriminative projecting, we build the *k*-nearest neighbor intraclass graph ${\left\{G_{\;}^{m}\right\}}_{m=1}^{M}$ and interclass graph ${\left\{P_{\;}^{m}\right\}}_{m=1}^{M}$ in each view, which use the supervised information to describe the local geometrical structure of the data. The intraclass graph $G_{\;}^{m}$ can be computed as

(5) $$\begin{array}{l} G_{i,j}^{m}=\{\begin{array}{c} {\;}^{1}\text{​​}╱\text{​}{\text{​}}_{\left|X_{s}\right|}\;if\;x_{i}^{m}\in N_{k_{1}}(x_{j}^{m})\;or\;x_{j}^{m}\in N_{k_{1}}(x_{i}^{m})\;and\;(x_{i}^{\;},x_{j}^{\;})\;\in X_{s},\\ \text{0 }otherwise \end{array} \end{array}$$

where $N_{k_{1}}\left(x_{j}^{m}\right)$ denotes the intraclass sample set containing the *k*_1_ nearest neighbors of $\;x_{i}^{m}$.

The graph $P_{\;}^{m}$ can be computed as

(6) $$\begin{array}{l} P_{i,j}^{m}=\{\begin{array}{c} {\;}^{1}\text{​​}╱\text{​}{\text{​}}_{\left|X_{d}\right|}\;if\;x_{i}^{m}\in N_{k_{2}}(x_{j}^{m})\;or\;x_{j}^{m}\in N_{k_{2}}(x_{i}^{m})\;and\;(x_{i}^{\;},x_{j}^{\;})\;\in X_{d},\\ \text{0 }otherwise \end{array} \end{array}$$

where $N_{k_{2}}\left(x_{j}^{m}\right)$ denotes the interclass sample set containing the *k*_2_ nearest neighbors of $\;x_{i}^{m}$.

Then, the intraclass correlation constraint $F_{G}^{m}$ from the *m*th view can be written as

(7) $$\begin{array}{l} \begin{array}{l} F_{G}^{m}=\frac{1}{2}\overset{N}{\underset{i,j}{\sum }}G_{i,j}^{m}\|H^{T}x_{i}^{m}-H^{T}x_{j}^{m}{\|}_{2}^{2}\\ \;=\frac{1}{2}\overset{N}{\underset{i,j}{\sum }}P_{i,j}^{m}\left(H^{T}\left(x_{i}^{m}-x_{j}^{m}\right){\left(x_{i}^{m}-x_{j}^{m}\right)}^{T}H\right)\\ \;=Tr\left(H^{T}X^{m}L_{G}^{m}X^{mT}H\right) \end{array} \end{array}$$

where $L_{G}^{m}$ is the Laplacian matrix on $G_{\;}^{m}$, and $L_{G}^{m}$ is computed as $L_{G}^{m}=D_{G}^{m}-G_{\;}^{m}$. $D_{G}^{m}$ is a diagonal matrix, and the element in $D_{G}^{m}$ is $D_{G,i,i}^{m}=\sum_{j}G_{i,j}^{m}$. *Tr* () is for the trace operator.

The interclass correlation constraint $F_{P}^{m}$ from the *m*th view can be written as

(8) $$\begin{array}{l} \begin{array}{l} F_{P}^{m}=\frac{1}{2}\overset{N}{\underset{i,j}{\sum }}P_{i,j}^{m}\|H^{T}x_{i}^{m}-H^{T}x_{j}^{m}{\|}_{2}^{2}\\ \;=\frac{1}{2}\overset{N}{\underset{i,j}{\sum }}P_{i,j}^{m}\left(H^{T}\left(x_{i}^{m}-x_{j}^{m}\right){\left(x_{i}^{m}-x_{j}^{m}\right)}^{T}H\right)\\ \;=Tr\left(H^{T}X^{m}L_{P}^{m}X^{mT}H\right) \end{array} \end{array}$$

where $L_{P}^{m}$ is the Laplacian matrix on $P_{\;}^{m}$, and $L_{P}^{m}$ is computed as $L_{P}^{m}=D_{P}^{m}-P_{\;}^{m}$. $D_{P}^{m}$ is a diagonal matrix, and the element in $D_{P}^{m}$ is $D_{p,i,i}^{m}=\sum_{j}P_{i,j}^{m}$.

For global structure knowledge of multiple-view data preservation, the following global structure consistency $Q_{\;}^{m}$ is employed

(9) $$\begin{array}{l} \begin{array}{l} Q_{\;}^{m}=\frac{1}{2}\overset{N}{\underset{i,j}{\sum }}W_{i,j}^{\;}\|H^{T}x_{i}^{m}-H^{T}x_{j}^{m}{\|}_{2}^{2}\\ \;=\frac{1}{2}\overset{N}{\underset{i,j}{\sum }}W_{i,j}^{\;}\left(H^{T}\left(x_{i}^{m}-x_{j}^{m}\right){\left(x_{i}^{m}-x_{j}^{m}\right)}^{T}H\right)\\ \;=Tr\left(H^{T}X^{m}L_{W}^{\;}X^{mT}H\right) \end{array} \end{array}$$

where ***W*** is an adjacent matrix whose element is $W_{i,j}^{\;}=1/N^{2}$. $L_{W}^{\;}$ is the Laplacian matrix on ***W***, and $L_{W}^{\;}$ is computed as $L_{W}^{\;}=D_{W}^{\;}-W$, $D_{W}^{\;}$ is a diagonal matrix, and the element in $D_{W}^{\;}$ is $D_{W,i,i}^{m}=1/N$.

The basic principle of $Q_{\;}^{m}$ is to use the global structural information through cross-view data covariance. The term $X^{m}L_{W}^{\;}X^{mT}$ is equivalent to the centering matrix of the *m*th view data, i.e., $X^{m}L_{W}^{\;}X^{mT}=E\left[\left(x-\frac{1}{N}\underset{i}{\sum }x_{i}\right){\left(x-\frac{1}{N}\underset{i}{\sum }x_{i}\right)}^{T}\right]$. It represents the average squared distance between all samples of the *m*th view in the metric space. Therefore, $Q_{\;}^{m}$ can be considered as a principal component analysis (PCA) (Smola, 1997)—like the regularization term in the *m*th view.

The goal of AMDML is to find an optimal discriminative distance metric in a multi-view learning model, and in such metric space, it can exploit the complementary information of different view data features and further enforce the proposed method to be more discriminative. To achieve this goal, we learn a metric that maximizes the Fisher analysis constraint (interclass/intraclass correlation ratio), simultaneously maximizing the preservation of the global structure consistency constraint. The objective function of AMDML is designed as

(10) $$\begin{array}{l} \begin{array}{l} \underset{H,\Theta }{\min}\overset{M}{\underset{m=1}{\sum }}\Theta_{m}^{r}\frac{F_{G}^{m}-F_{P}^{m}}{Q_{\;}^{m}},\\ s.t.\;H^{T}H=I,\\ \;\overset{M}{\underset{m=1}{\sum }}\Theta_{m}=1,\;\Theta_{m}\geq 0, \end{array} \end{array}$$

The projection matrix ***H*** helps to build a discriminative metric space among multiple views, such that the feature correlation and complementary structural information among multiple views can be exploited. The vector ***Θ*** = [Θ_1_, Θ_2_, ..., Θ_*M*_] is the view weight vector, and its element Θ_*m*_ indicates the role importance of the *m*th view. When Θ_*m*_ tends to 0, it means the data features of the *m*th view are useless for discrimination task. The Θ_*m*_=1 means that only one type of data features from one view is used in AMDML, and in this case, Equation (10) is a single view learning problem. To better utilize the complementary information of multiple features rather than the best feature, we use index parameter *r* (*r* > 1) on Θ_*m*_.

Equation (10) can be represented as

(11) $$\begin{array}{l} \begin{array}{l} \underset{H,\Theta }{\min}\overset{M}{\underset{m=1}{\sum }}\Theta_{m}^{r}\frac{Tr\left(H^{T}X^{m}\left(L_{G}^{m}-L_{P}^{m}\right)X^{mT}H\right)}{Tr\left(H^{T}X^{m}L_{W}^{\;}X^{mT}H\right)},\\ s.t.\;H^{T}H=I,\\ \;\overset{M}{\underset{m=1}{\sum }}\Theta_{m}=1,\;\Theta_{m}\geq 0. \end{array} \end{array}$$

However, the optimization of Equation (11) involves a complex operation of inverse. Using a constraint weight parameter γ, we reconstruct Equation (11) into the following weighted optimization model

(12) $$\begin{array}{l} \begin{array}{l} \underset{H,\Theta ,\gamma }{\min}\overset{M}{\underset{m=1}{\sum }}\Theta_{m}^{t}\left(\gamma^{2}H^{T}X^{m}\left(L_{G}^{m}-L_{P}^{m}\right)X^{mT}H-\gamma Tr\left(H^{T}X^{m}L_{W}^{\;}X^{mT}H\right)\right),\\ s.t.\;H^{T}H=I,\\ \;\overset{M}{\underset{m=1}{\sum }}\Theta_{m}^{\;}=1,\;\Theta_{m}^{\;}\geq 0. \end{array} \end{array}$$

where γ represents a constraint weight tradeoff Fisher discriminative constraint and global structure constraint. It is noted that the constraint weight γ and view weight Θ are not a manually adjusted parameters. In this study, we adaptively adjust γ and Θ in two closed form solutions, respectively.

### Optimization

Because the optimization problem of Equation (12) is a non-linear constrained non-convex problem, in this study, we solve the optimization problem in Equation (12) using the iteratively optimization strategy to obtain the AMDML parameters of ***H***, ***Θ***, and γ. First, we tune parameter ***H*** while fixing parameters ***Θ*** and γ. The optimization problem in Equation (12) can be reformulated as follows:

(13) $$\begin{array}{l} \begin{array}{l} \underset{H}{\min}\overset{M}{\underset{m=1}{\sum }}\Theta_{m}^{t}\left(\gamma^{2}Tr\left(H^{T}X^{m}\left(L_{G}^{m}-L_{P}^{m}\right)X^{mT}H\right)-\gamma Tr\left(H^{T}X^{m}L_{W}^{\;}X^{mT}H\right)\right),\\ s.t.\;H^{T}H=I, \end{array} \end{array}$$

Thus, ***H*** can be easily calculated by solving the eigenvalue decomposition problem as follows:

(14) $$\begin{array}{l} \left(\overset{M}{\underset{m=1}{\sum }}\Theta_{m}^{t}\left(\gamma^{2}X^{m}\left(L_{G}^{m}-L_{P}^{m}\right)X^{mT}-\gamma \left(X^{m}L_{W}^{\;}X^{mT}\right)\right)\right)H=\alpha H \end{array}$$

In terms of the Lagrange optimization, the minimization of Equation (14) can be converted with multiplier as follows:

(15) $$\begin{array}{l} J(\Theta ,\gamma ,\alpha )=\sum_{m=1}^{M}\Theta_{m}^{t}(\gamma^{2}Tr(H^{T}X^{m}(L_{G}^{m}-L_{P}^{m})X^{mT}H)\\ \;-\gamma Tr(H^{T}X^{m}L_{W}^{\;}X^{mT}H))\;-\alpha (\sum_{m=1}^{M}\Theta_{m}^{t}-1). \end{array}$$

Next, we tune parameter ***Θ*** while fixing parameters ***H*** and γ.

Let $\frac{\partial J\left(\Theta ,\gamma ,\alpha \right)}{\partial \Theta_{m}^{\;}}=0$ and $\frac{\partial J\left(\Theta ,\gamma ,\alpha \right)}{\partial \alpha }=0$, we have

(16) $$\begin{array}{l} \{\begin{array}{l} t\Theta_{m}^{t-1}\gamma^{2}(Tr(H^{T}X^{m}(L_{G}^{m}-L_{P}^{m})X^{mT}H)-\gamma Tr(H^{T}X^{m}L_{W}^{\;}X^{mT}H))-\alpha =0,\\ \sum_{m=1}^{M}\Theta_{m}-1=0. \end{array} \end{array}$$

We can obtain Θ_*m*_ as follows:

(17) $$\begin{array}{l} \Theta_{m}=\frac{{\left(1/\left(\gamma^{2}Tr\left(H^{T}X^{m}\left(L_{G}^{m}-L_{P}^{m}\right)X^{mT}H\right)-\gamma Tr\left(H^{T}X^{m}L_{W}^{\;}X^{mT}H\right)\right)\right)}^{1/\left(t-1\right)}}{\overset{M}{\underset{m=1}{\sum }}{\left(1/\left(\gamma^{2}Tr\left(H^{T}X^{m}\left(L_{G}^{m}-L_{P}^{m}\right)X^{mT}H\right)-\gamma Tr\left(H^{T}X^{m}L_{W}^{\;}X^{mT}H\right)\right)\right)}^{{\;}^{1/\left(t-1\right)}}}. \end{array}$$

Finally, we tune parameter γ while fixing parameters ***H*** and ***Θ***. In terms of the Lagrange optimization, the solution of γ is $\frac{\partial J\left(\Theta ,\gamma ,\alpha \right)}{\partial \gamma }=0$; we can obtain γ as follows:

(18) $$\begin{array}{l} \gamma =\frac{\Theta_{m}^{t}\overset{M}{\underset{m=1}{\sum }}Tr\left(H^{T}X^{m}L_{W}^{\;}X^{mT}H\right)}{2\Theta_{m}^{t}\overset{M}{\underset{m=1}{\sum }}Tr\left(H^{T}X^{m}\left(L_{G}^{m}-L_{P}^{m}\right)X^{mT}H\right)} \end{array}$$

Based on the above analysis, the proposed AMDML method is presented in **Algorithm 1**.

**Algorithm 1:** The proposed AMDML method.

**Input:** M views of *m* pairs of EEG signals;
**Output:** the best metric *H* = *H*_*l*_.
Set **Θ** = [1/*M*, 1/*M*, ..., 1/*M*] and compute ***H*** using **Equation (13)**.
Repeat
*t* = *t* + 1
Fix ***H***(*t*), and compute **Θ**(*t*) using **Equation (17)**;
Fix **Θ**(*t*), and compute ***H***(*t*) using **Equation (18)**
Compute *L*(t) using **Equation (15)**;
Until ||*J*(t)-*J*(t-1)|| ≤ δ or *t* ≥ *t*_max_

## Experiment

### Experimental Settings

In the experiment, we extract three types of data feature including KPCA, wavelet packet decomposition (WPD) (Wu et al., 2008), and short-time Fourier transform (STFT) (Griffin and Lim, 1984). We design 10 classification tasks, and the basic information of tasks is as shown in Table 2. In order to show the performance of our method, we compare AMDML with four single-view classification methods [including DMSI (Xing et al., 2003), large margin nearest neighbor (LMNN) (Weinberger and Saul, 2009), neighborhood preserving embedding (NPE) (Wen et al., 2010), and RDML-CCPVL (Ni et al., 2018)] and three multi-view methods [including MvCVM (Huang et al., 2015), VMRML-LS (Quang et al., 2013), and DMML (Zhang et al., 2019)]. In the LMNN method, the number of target neighbors *k* was set to *k* = 3, and the weighting parameter μ is selected within the grid {0, 0.2,., 1}. In the RDML-CCPVL method, the regularization parameter is selected within the grid [0.01, 0.1, 0.5, 1, 5, 10, 20] and the number of clusters is selected within the grid [2, 3,., 20]. In the MvCVM method, the regularization parameter is selected within the grid [0.01, 0.1, 0.5, 1, 5, 10, 20]. In VMRML-LS, the regularization parameters $\gamma_{A}=10^{-5}$, $\gamma_{B}=10^{-6}$, and $\gamma_{W}=10^{-6}$, and the element in weight vector is selected in [1, 5, 10]. In the DMML method, the number of interclass marginal samples is selected within the grid [1, 2,., 10]. In the proposed AMDML method, the parameters *k*_1_ and *k*_2_ in **Equations (5)** and **(6)** are selected in [2, 3,., 10]. The widely used *K*-nearest neighbor (KNN) and support vector machine (SVM) are used as the classifiers for the proposed AMDML, and we name them as AMDML-KNN and AMDML-SVM, respectively. We empirically set the nearest neighborhood number of KNN classifier as [1, 3,., 9] and train SVM model using LIBSVM (Chang and Lin, 2011). All methods are implemented in MATLAB using a computer with 2.6 GHz dual-core CPU and 8 GB RAM.

**TABLE 2:** Ten EEG classification tasks.

**Tasks**	**EEG signal groups**
Task 1	A and C
Task 2	A and D
Task 3	A and E
Task 4	B and C
Task 5	B and D
Task 6	B and E
Task 7	{A, B} and {C, D}
Task 8	{A, B} and E
Task 9	{A, B} and {D, E}
Task 10	{A, B} and {C, E}

### Performance Comparisons With Single-View Methods

We first compare the performance of AMDML with several single-view classification methods. NPE using two classifiers KNN and SVM are named as NPE-KNN and NPE-SVM, respectively. Table 3 shows the classification performance of these methods on Bonn EEG dataset using three signal views (WPD, STFT, and KPCA) and full views. When AMDML uses single-view feature data, the parameter Θ_*m*_ is fixed with Θ_*m*_=1 in its objective function. For a fair comparison, three signal views features are combined for four single-view classification methods in full views. We can see that, on the one hand, both AMDML-KNN and AMDML-SVM using full-view features are better than them using only single-view features. For example, the performances of AMDML-KNN with full-view feature are 1.44, 1.57, and 1.31% higher than its performance in WPD, STFT, and KPCA on Task 1, respectively. On the other hand, the classification accuracy of methods AMDML-KNN and AMDML-SVM are better than single-view methods on 10 tasks. These results show that (1) the simple combination of features is limited to improve classification performance for single-view methods, and (2) due to the inherent diversity and complex of EEG signals, it is suitable to exploit multiple view features to better make use of the correlation and complementary EEG data. Thus, the multi-view learning framework can promote the EEG signal classification performance.

**TABLE 3:** The classification performances of AMDML on 10 classification tasks.

		**WPD**	**STFT**	**KPCA**	**Full views**
Task 1	LMNN	95.00	96.14	96.19	96.18
	NPE-KNN	95.05	96.29	96.34	96.38
	NPE-SVM	95.08	96.17	96.29	96.49
	RDML-CCPVL	96.07	96.64	96.58	96.97
	DMSI	96.11	96.53	96.52	96.88
	AMDML-KNN	97.75	97.62	97.88	**99.19**
	AMDML-SVM	97.86	97.55	97.86	99.18
Task 2	LMNN	93.92	94.73	95.00	95.88
	NPE-KNN	94.01	95.03	95.21	95.71
	NPE-SVM	94.06	95.00	94.54	96.07
	RDML-CCPVL	94.60	95.06	95.88	96.17
	DMSI	94.45	95.08	94.64	96.22
	AMDML-KNN	97.29	97.86	98.38	99.77
	AMDML-SVM	97.22	98.03	98.30	**99.78**
Task 3	LMNN	94.75	94.92	95.11	95.40
	NPE-KNN	94.01	94.01	95.42	95.72
	NPE-SVM	94.98	94.08	95.41	95.64
	RDML-CCPVL	94.56	95.59	95.88	95.70
	DMSI	94.72	95.34	95.26	96.24
	AMDML-KNN	96.72	98.82	98.35	99.35
	AMDML-SVM	96.83	98.90	98.48	**99.41**
Task 4	LMNN	92.76	92.90	93.07	94.45
	NPE-KNN	94.00	93.02	94.48	94.70
	NPE-SVM	93.00	93.04	93.42	94.65
	RDML-CCPVL	93.52	93.56	93.90	94.92
	DMSI	94.21	94.01	94.45	95.03
	AMDML-KNN	96.71	97.88	97.44	**99.42**
	AMDML-SVM	96.88	97.86	97.42	99.35
Task 5	LMNN	91.81	93.89	94.10	95.46
	NPE-KNN	92.99	93.08	93.43	95.71
	NPE-SVM	92.98	93.13	94.46	95.66
	RDML-CCPVL	94.54	94.66	94.91	95.99
	DMSI	94.50	95.04	95.11	95.37
	AMDML-KNN	98.76	98.87	98.40	99.39
	AMDML-SVM	99.83	98.89	98.39	**99.41**
Task 6	LMNN	96.72	96.92	96.16	97.36
	NPE-KNN	96.05	96.93	97.09	97.22
	NPE-SVM	96.91	97.01	97.22	97.42
	RDML-CCPVL	96.57	97.62	96.90	97.37
	DMSI	96.49	97.49	96.81	97.02
	AMDML-KNN	97.73	98.89	97.39	**99.45**
	AMDML-SVM	97.89	98.94	97.35	99.38
Task 7	LMNN	93.76	93.87	93.05	95.37
	NPE-KNN	93.93	94.06	93.41	95.73
	NPE-SVM	96.03	95.97	95.43	95.68
	RDML-CCPVL	95.54	95.59	94.94	95.94
	DMSI	95.29	95.36	94.73	95.08
	AMDML-KNN	97.74	97.89	97.31	99.39
	AMDML-SVM	97.92	97.81	97.36	**99.44**
Task 8	LMNN	94.79	95.86	95.12	96.40
	NPE-KNN	94.95	96.00	95.44	96.41
	NPE-SVM	94.94	95.99	95.45	96.09
	RDML-CCPVL	95.57	96.09	95.89	96.14
	DMSI	95.63	95.94	96.15	96.18
	AMDML-KNN	97.72	98.82	98.41	99.45
	AMDML-SVM	97.90	98.92	98.45	**99.46**
Task 9	LMNN	92.78	94.94	94.06	95.37
	NPE-KNN	92.93	95.05	94.44	95.68
	NPE-SVM	93.01	95.04	95.43	95.62
	RDML-CCPVL	94.52	95.66	95.94	95.98
	DMSI	94.67	92.51	96.04	96.40
	AMDML-KNN	96.69	97.83	97.37	**99.42**
	AMDML-SVM	96.99	97.90	97.43	99.38
Task 10	LMNN	93.87	93.86	93.13	94.31
	NPE-KNN	94.02	92.97	93.44	94.49
	NPE-SVM	93.04	94.09	94.48	94.66
	RDML-CCPVL	95.57	95.56	94.98	96.01
	DMSI	95.17	94.22	94.10	94.56
	AMDML-KNN	97.78	97.83	96.41	**98.40**
	AMDML-SVM	97.89	97.97	96.38	98.37

*The bold values represents mean the best classification performance in the tasks*.

### Performance Comparisons With Multi-View Methods

**Figure 2:**
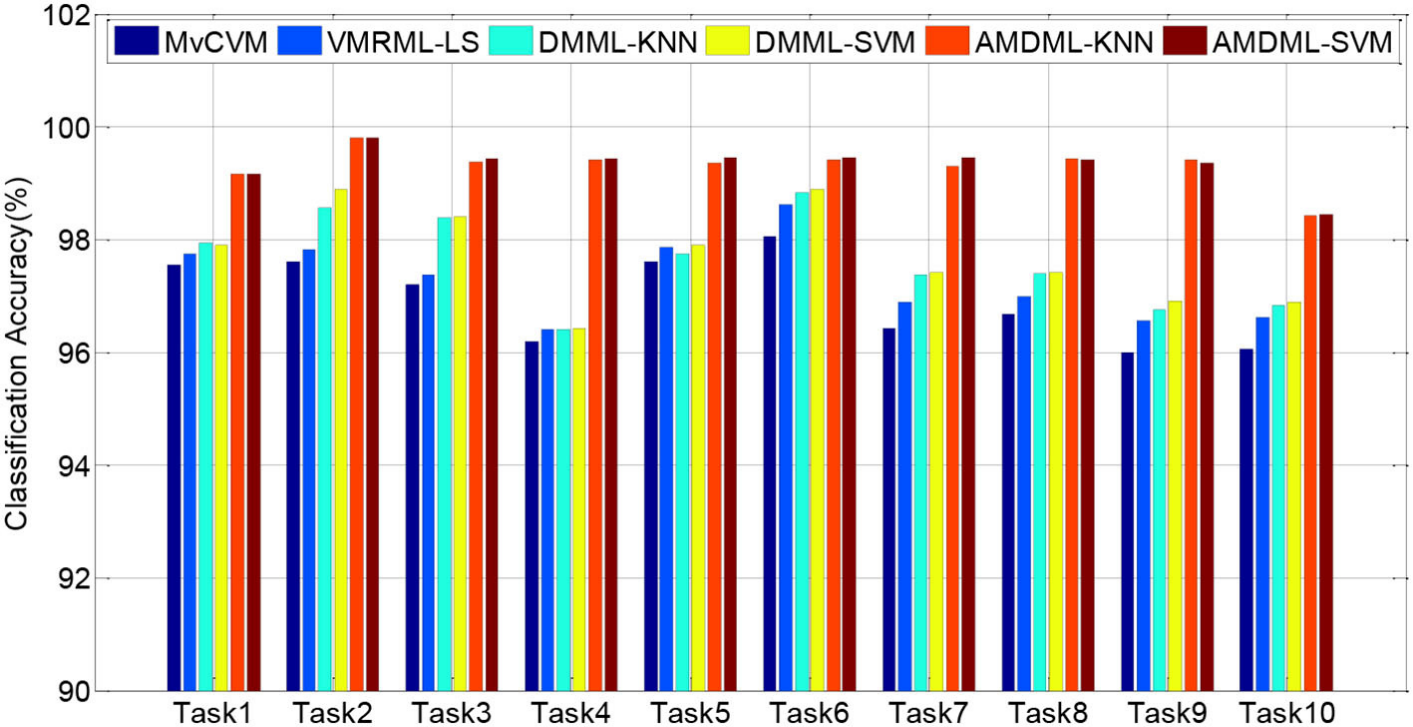
Classification accuracies of six methods on 10 EEG classification tasks.

In this subsection, we compare AMDML with several multi-view classification methods. The multi-view metric learning method DMML uses KNN and SVM as testing classifiers, and two classifiers are named as DMML-KNN and DMML-SVM, respectively. Figure 2 shows the classification accuracies of all methods on all EEG classification tasks. In addition, we use balanced loss *l*_*bal*_ (Wang et al., 2014; Gu et al., 2020) to evaluate the classification accuracy on positive class *ACC*_*positive*_ and negative class *ACC*_*negative*_:

$$\begin{array}{l} l_{bal}=1-\left(ACC_{positive}+ACC_{negative}\right)/2 \end{array}$$

**Figure 3:**
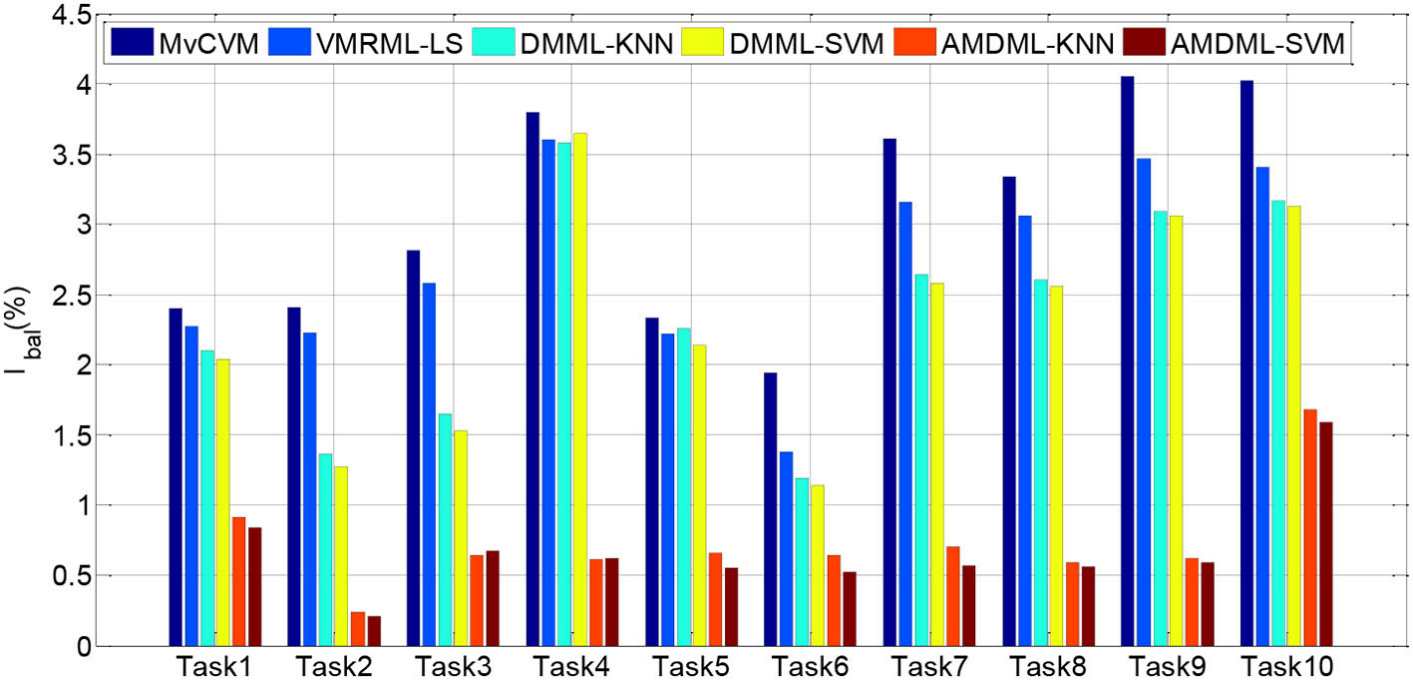
*l*_*bal*_ performance of six methods on all EEG classification tasks.

Figure 3 shows *l*_*bal*_ performance of all methods on EEG classification tasks. Experiment results show that compared with all multi-view classification methods, both AMDML-KNN and AMDML-SVM have a positive effect on improving classification performance. AMDML-KNN and AMDML-SVM achieve the satisfactory classification performance in almost all of the EEG signal categories. In the framework of multi-view learning and to discriminate each emotion category best from all other categories, AMDML learns discriminative metric space to utilize the global and local information by adopting Fisher discriminative constraint and global structure constraint. Thus, the intraclass compactness and interclass EEG signals separability can perform better in the learned metric space. In addition, the auto-weighted learning strategy used in the proposed method adjusts constraint and view weights. The optimal weights can be obtained adaptively, and multiple feature representation in each view can be collaborative leaned. Similar to the results shown in Table 3, the classification accuracies of AMDML-KNN and AMDML-SVM are comparative. To summarize, the results in Figures 2, 3 confirm that the AMDML method is effective in EEG signal classification.

### Model Analysis

**Figure 4:**
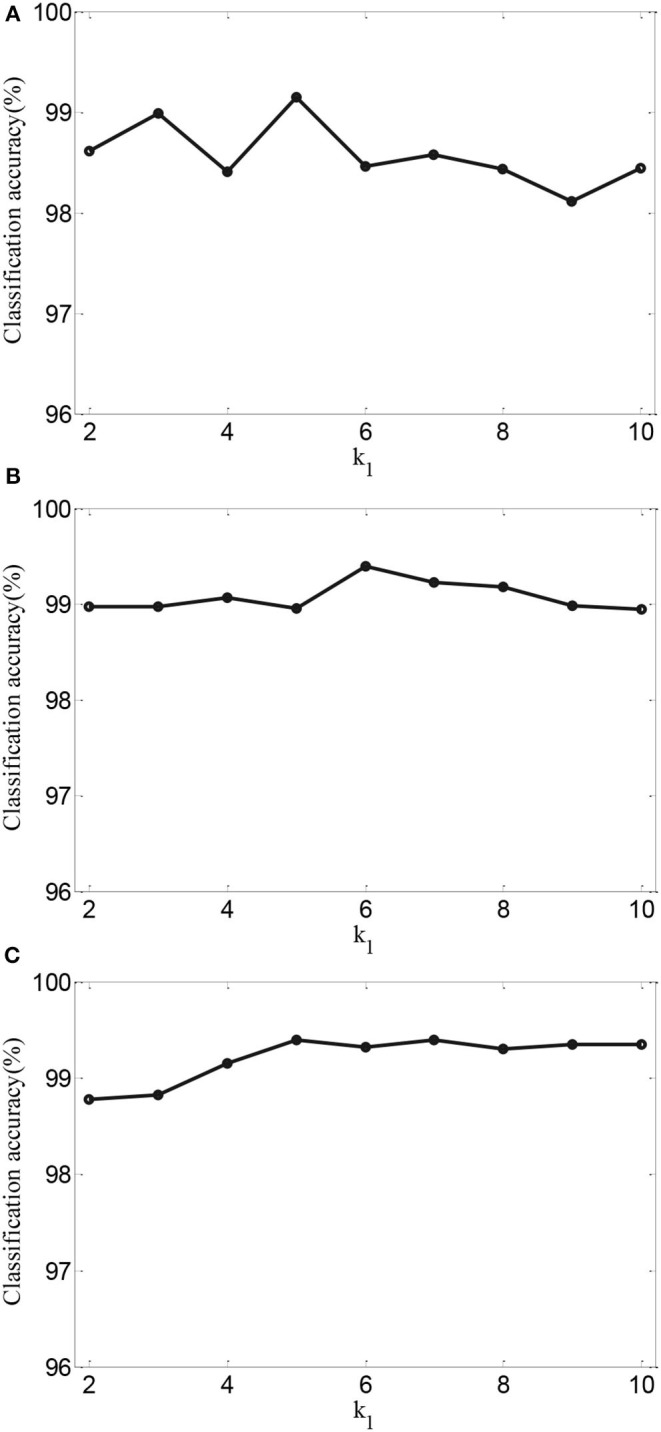
Classification accuracy of AMDML-KNN with different values of *k*_1_ for **(A)** Task 1, **(B)** Task 4, and **(C)** Task 8.

To further validate the effects of performance of AMDML, we discuss the effect of the *k*_1_ in **Equation (5)** and *k*_2_ in **Equation (6)** in AMDML. The parameters *k*_1_ and *k*_2_ build the *k*-nearest neighbor inter- and intraclass graphs, respectively. For convenience, we set *k*_1_ = *k*_2_ in the range {2,., 10}. Figure 4 shows the classification accuracy of AMDML-KNN with different values of *k*_1_ for Tasks 1, 4, and 8; meanwhile, the *k*-nearest neighbor in KNN are fixed with 7. We can see that the performance of AMDML-KNN is not high sensitive to the variation *k*_1_ and *k*_2_.

**Figure 5:**
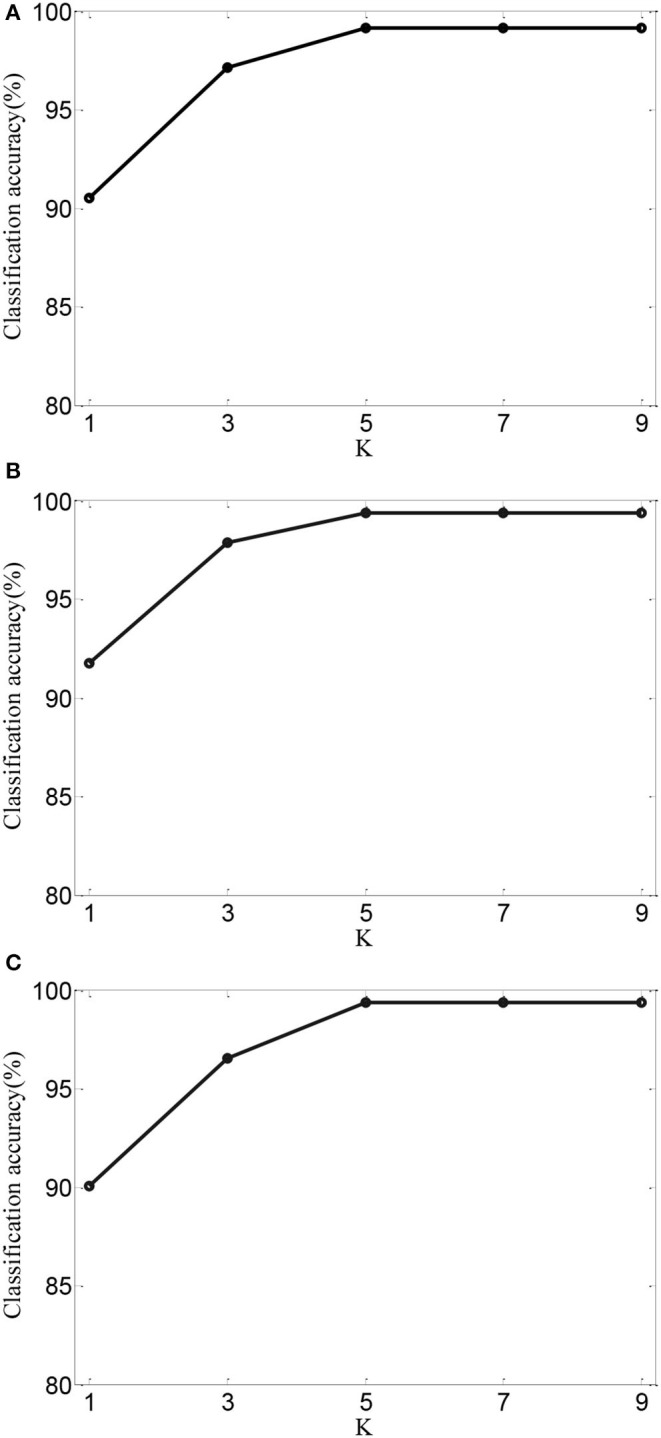
Classification accuracy of AMDML-KNN with different values of *K* for **(A)** Task 1, **(B)** Task 4, and **(C)** Task 8.

Next, for AMDML-KNN, we discuss the effect of the *K* in KNN classifier. In KNN classifier, the class label of the testing sample is determined by the distance from the *K* nearest training sample. Figure 5 shows the classification performance of different values of *K* for all tasks; meanwhile, fixing *k*_1_ = *k*_2_ = 5. We can see that the classification accuracy of AMDML-KNN is relatively stable with respect to the variation *K*. Therefore, we can set *K* empirically to 7 for Tasks 1, 4, and 8.

## Conclusion

In this paper, we propose a new multi-views metric learning to achieve the robust distance metric for EEG signal classification. In the scheme of the multi-view data representation, the diversity, and complementariness of features of all views can be exploited; meanwhile, both the Fisher discriminative constraint and global structure constraint are considered, and the learned classifier will obtain high generalization ability. Through learning a discriminative metric space, AMDML shows the higher classification performance. There are several directions of future study. In this paper, we use the *k*-nearest neighbor intra- and interclass graphs to exploit local discriminative information; we will consider other discriminative terms in the multi-view metric learning framework. Second, the gradient descent method used in this study is a simple and common solution method. We may develop a more effective method to speed up the solution of our method. Third, we plan to apply the proposed method for more EEG signal classification applications.

## Data Availability Statement

Publicly available datasets were analyzed in this study. This data can be found here: http://epileptologie-bonn.de/cms/upload/workgroup/lehnertz/eegdata.html.

## Author Contributions

JX and TN conceived and developed the theoretical framework of the manuscript. XG performed the data process, data evaluation, analysis, interpretation, and designed the figures. All authors drafted the manuscript.

## Conflict of Interest

The authors declare that the research was conducted in the absence of any commercial or financial relationships that could be construed as a potential conflict of interest.

## References

[B1] Alwasiti H. Yusoff M. Z. Raza K. (2020). Motor imagery classification for brain computer interface using deep metric learning. IEEE Access 8, 109949–109963. 10.1109/ACCESS.2020.3002459 PMC9788676 36578777

[B2] Cai D. Liu K. Su F. (2015). “Local metric learning for EEG-based personal identification,” in Proceedings of 2015 IEEE International Conference on Acoustics, Speech and Signal Processing (ICASSP) (Brisbane, QLD), 842–846. 10.1109/ICASSP.2015.7178088

[B3] Chang C. C. Lin C. J. (2011). LIBSVM: a library for support vector machines. ACM Trans. Intelligent Intellig. Syst. Technol. 2, 1–27. 10.1145/1961189.1961199

[B4] Cury C. Maurel P. Gribonval R. Barillot C. (2019). A sparse EEG-informed fMRI model for hybrid EEG-fMRI neurofeedback prediction. Front. Neurosci. 13:1451. 10.3389/fnins.2019.01451 32076396 PMC7006471

[B5] Griffin D. Lim J. S. (1984). Signal estimation from modified short-time fourier transform. IEEE Trans. Acoustics Speech Signal Process. 32, 236–243. 10.1109/TASSP.1984.1164317

[B6] Gu X. Chung K. Wang S. (2020). Extreme vector machine for fast training on large data. Int. J. Mach. Learn. Cybernet. 11, 33–53. 10.1007/s13042-019-00936-3

[B7] Gummadavelli A. Zaveri H. P. Spencer D. D. Gerrard J. L. (2018). Expanding brain-computer interfaces for controlling epilepsy networks: novel thalamic responsive neurostimulation in refractory epilepsy. Front. Neurosci. 12:474. 10.3389/fnins.2018.00474 30108472 PMC6079216

[B8] Huang C. Chung F. L. Wang S. (2015). Multi-view L2-svm and its multi-view core vector machine. Neural Netw. . 75, 110–125. 10.1016/j.neunet.2015.12.004 26773824

[B9] Iscan Z. Dokur Z. Demiralp T. (2011). Classification of electroencephalogram signals with combined time and frequency features. Expert Syst. Appl. 38, 10499–10505. 10.1016/j.eswa.2011.02.110

[B10] Jiang Y. Deng Z. Chung F. Wang G. Qian P. Choi K. S. (2017b). Recognition of epileptic EEG signals using a novel multiview TSK fuzzy system. IEEE Trans. Fuzzy Syst. 25, 3–20. 10.1109/TFUZZ.2016.2637405 28880184

[B11] Jiang Y. Wu D. Deng Z. Qian P. Wang J. Wang G.. (2017a). Seizure classification from EEG signals using transfer learning, semi-supervised learning and TSK fuzzy system. IEEE Trans. Neural Syst. Rehabil. Eng. 25, 2270–2284. 10.1109/TNSRE.2017.2748388 28880184

[B12] Juan D. M. V. Gregor S. Kristl V. Pieter M. German C. Dominguez. (2017). Improved localization of seizure onset zones using spatiotemporal constraints and time-varying source connectivity. Front. Neurosci. 11:156. 10.3389/fnins.2017.00156 28428738 PMC5382162

[B13] Kabir E. Zhang Y. (2016). Epileptic seizure detection from EEG signals using logistic model trees. Brain Inform. 3, 93–100, 10.1007/s40708-015-0030-2 27747604 PMC4883168

[B14] Li X. Yang H. Yan J. Wang X. Li X. Yuan Y. (2019). Low-intensity pulsed ultrasound stimulation modulates the nonlinear dynamics of local field potentials in temporal lobe epilepsy. Front. Neurosci. 13:287. 10.3389/fnins.2019.00287 31001072 PMC6454000

[B15] Liu Q. Zhao X. Hou Z. (2014). “Metric learning for event-related potential component classification in EEG signals,” in Proceedings of 2014 22nd European Signal Processing Conference (EUSIPCO) (Lisbon), 2005–2009.

[B16] Lu X. Wang Y. Zhou X. Ling Z. (2016). A method for metric learning with multiple-kernel embedding. Neural Process. Lett. 43, 905–921. 10.1007/s11063-015-9444-3

[B17] Ni T. Ding Z. Chen F. Wang H. (2018). Relative distance metric leaning based on clustering centralization and projection vectors learning for person Re-identification. IEEE Access 6, 11405–11411. 10.1109/ACCESS.2018.2795020

[B18] Phan H. Do Q. Do T. L. Vu D. L. (2013). “Metric learning for automatic sleep stage classification,” in Proceedings of 2013 35th Annual International Conference of the IEEE Engineering in Medicine and Biology Society (EMBC) (Osaka), 5025–5028. 10.1109/EMBC.2013.6610677 24110864

[B19] Quang M. H. Bazzani L. Murino V. (2013). “A unifying framework for vector-valued manifold regularization and multi-view learning,” in Proceedings of the 30th International Conference on International Conference on Machine Learning (Atlanta, GA), 100–108.

[B20] Richhariya B. Tanveer M. (2018). EEG signal classification using universum support vector machine. Expert Syst. Appl. 106, 169–182. 10.1016/j.eswa.2018.03.053

[B21] Smola A. J. (1997). “Kernel principal component analysis,” in Proceedings of International Conference on Artificial Neural Networks (Lausanne), 583–588. 10.1007/BFb0020217

[B22] Spyrou L. Kouchaki S. Sanei S. (2018). Multiview classification and dimensionality reduction of scalp and intracranial EEG data through tensor factorisation. J. Signal Process. Syst. 90, 273–284. 10.1007/s11265-016-1164-z

[B23] Srinivasan V. Eswaran C. Sriraam N. (2005). Artificial neural network based epileptic detection using time-domain and frequency-domain features. J. Med. Syst. 29, 647–660. 10.1007/s10916-005-6133-1 16235818

[B24] Tzallas A. T. Tsipouras M. G. Fotiadis I. D. (2009). Epileptic seizure detection in EEGs using time-frequency analysis. IEEE Trans. Inform. Technol. Biomed. 13, 703–710. 10.1109/TITB.2009.2017939 19304486

[B25] Usman S. M. Fong S. (2017). Epileptic seizures prediction using machine learning methods. Computat. Math. Methods Med. 2017:9074759. 10.1155/2017/9074759 29410700 PMC5749318

[B26] Wang F. Zuo W. Zhang L. Meng D. Zhang D. (2015). A kernel classification framework for metric learning, IEEE Trans. Neural Netw. Learn. Syst. 26, 1950–1962. 10.1109/TNNLS.2014.2361142 25347887

[B27] Wang S. Wang J. Chung K. (2014). Kernel density estimation, kernel methods, and fast learning in large data sets. IEEE Trans. Cybernet. 44, 1–20. 10.1109/TSMCB.2012.2236828 23797315

[B28] Weinberger K. Q. Saul L. K. (2009). Distance metric learning for large margin nearest neighbor classification. Mach. Learn. Res. 10, 207–244.

[B29] Wen D. Li P. Zhou Y. Sun Y. Xu J. Liu Y.. (2020). Feature classification method of resting-state EEG signals from amnestic mild cognitive impairment with type 2 diabetes mellitus based on multi-view convolutional neural network. IEEE Trans. Neural Syst. Rehabil. Eng. 28, 1702–1709. 10.1109/TNSRE.2020.3004462 32746302

[B30] Wen J. Tian Z. She H. Yan W. (2010). “Feature extraction of hyperspectral images based on preserving neighborhood discriminant embedding,” in Proceedings of IEEE Conference: Image Analysis and Signal Processing (IASP) (Xiamen), 257–262.

[B31] Wu T. Yan G. Z. Yang B. H. Hong S. (2008). EEG feature extraction based on wavelet packet decomposition for brain computer interface. Measurement 41, 618–625. 10.1016/j.measurement.2007.07.007

[B32] Xing E. P. Jordan M. I. Russell S. J. Ng A. Y. (2003). “Distance metric learning with application to clustering with side-information,” in Advances in Neural Information Processing Systems, (Cambridge, MA, USA: MIT Press), 521–528.

[B33] Yuan Y. Xun G. Jia K. Zhang A. (2019). A multi-view deep learning framework for EEG seizure detection. IEEE J. Biomed. Health Inform. 23, 83–94. 10.1109/JBHI.2018.2871678 30624207

[B34] Zhang L. Shum H. P. H. Liu L. Guo G. Shao L. (2019). Multiview discriminative marginal metric learning for makeup face verification. Neurocomputing 333, 339–350. 10.1016/j.neucom.2018.12.003

[B35] Zhou Z. Zhang Y. Jiang Y. (2019). “Deep view-reduction TSK fuzzy system: a case study on epileptic EEG signals detection,” in Proceedings of 2019 IEEE Symposium Series on Computational Intelligence (SSCI) (Xiamen), 387–392. 10.1109/SSCI44817.2019.9002722

